# Effectiveness of online and mobile telephone applications (‘apps’) for the self-management of suicidal ideation and self-harm: a systematic review and meta-analysis

**DOI:** 10.1186/s12888-017-1458-0

**Published:** 2017-08-15

**Authors:** Katrina Witt, Matthew J. Spittal, Gregory Carter, Jane Pirkis, Sarah Hetrick, Dianne Currier, Jo Robinson, Allison Milner

**Affiliations:** 1 Population Health, Turning Point, Eastern Health Clinical School, Monash University, 54-62 Gertrude Street, Fitzroy, Victoria 3065 Australia; 2 Melbourne School of Population and Global Health, University of Melbourne, Melbourne, Victoria Australia; 3 Centre for Translational Neuroscience and Mental Health, Faculty of Health and Medicine, University of Newcastle, Callaghan, New South Wales Australia; 4 Orygen, the National Centre of Excellence in Youth Mental Health and the Centre for Youth Mental Health, University of Melbourne, Melbourne, Victoria Australia; 5 Centre for Health Equity, Melbourne School of Population and Global Health, University of Melbourne, Melbourne, Victoria Australia

**Keywords:** Digital, Application, Mobile telephone, Suicide ideation, Self-harm, Suicide

## Abstract

**Background:**

Online and mobile telephone applications (‘apps’) have the potential to improve the scalability of effective interventions for suicidal ideation and self-harm. The aim of this review was therefore to investigate the effectiveness of digital interventions for the self-management of suicidal ideation or self-harm.

**Methods:**

Seven databases (Applied Science & Technology; CENTRAL; CRESP; Embase; Global Health; PsycARTICLES; PsycINFO; Medline) were searched to 31 March, 2017. Studies that examined the effectiveness of digital interventions for suicidal ideation and/or self-harm, or which reported outcome data for suicidal ideation and/or self-harm, within a randomised controlled trial (RCT), pseudo-RCT, or observational pre-test/post-test design were included in the review.

**Results:**

Fourteen non-overlapping studies were included, reporting data from a total of 3,356 participants. Overall, digital interventions were associated with reductions for suicidal ideation scores at post-intervention. There was no evidence of a treatment effect for self-harm or attempted suicide.

**Conclusions:**

Most studies were biased in relation to at least one aspect of study design, and particularly the domains of participant, clinical personnel, and outcome assessor blinding. Performance and detection bias therefore cannot be ruled out. Digital interventions for suicidal ideation and self-harm may be more effective than waitlist control. It is unclear whether these reductions would be clinically meaningful at present. Further evidence, particularly with regards to the potential mechanisms of action of these interventions, as well as safety, is required before these interventions could recommended.

## Background

Self-harm, which includes intentional self-injury or self-poisoning irrespective of type of motivation and/or degree of suicidal intent [1], and attempted suicide, which refers to any intentionally self-inflicted self-injurious and/or self-poisoning behaviour with clear suicidal intent [2, 3], are associated with suicidal ideation [4], non-fatal repetition of self-harm, and completed suicide [5]. Although in the United States of America (USA) distinction is frequently made between non-suicidal self-injury and suicidal behavior [6], outside of the USA these terms are yet to receive widespread acceptance [7]. Instead, this review includes all forms of self-inflected self-injury or self-poisoning, irrespective of type of motivation or degree of suicidal intent, which we refer to collectively as ‘self-harm’ [8].

Effective face-to-face treatments for self-harm and suicidal ideation are available [9–11]. Many effective psychotherapeutic options for the treatment of suicidal ideation and self-harm are resource intensive and require specialist clinician training, however. Resource limitations in some low-to-middle income countries therefore limits access to these professionals. Negative associations have, for example, been found between per capita availability of mental health services and suicide rates in a number of countries [12–14].

Even in countries where access to psychotherapy for suicidal ideation and self-harm are available, less than one-half of those who self-harm receive treatment [15]. There are a number of barriers to treatment for those who experience self-harm or suicidal ideation, including: beliefs that treatment is not warranted and/or is likely to be ineffective, stigma, shame, negative prior experiences with mental health care providers, and financial difficulties [15]. Young people in particular also cite a preference for self-management as a major obstacle to help-seeking for self-harm from clinical services [16].

Given that an estimated 85% of the global population is covered by a commercially-available wireless signal, and further, over five billion persons have a mobile phone subscription [17], digital interventions, including both online programs and mobile telephone applications (‘apps’) (collectively referred to here as ‘digital interventions’), have been proposed as one mechanism by which the scalability of effective treatments for self-harm and suicidal ideation may be improved [18]. Such interventions may also help to overcome some of the attitudinal and structural barriers which prevent those who engage in self-harm from accessing clinical services [19], and may therefore represent a valuable addition to a stepped care treatment model in which access is improved through the provision of lower intensity ‘self-help’ interventions in addition to high intensity psychotherapeutic treatments for those whose symptoms do not resolve.

To date, the effectiveness of these digital interventions has not been routinely evaluated. This is problematic given that the widespread implementation of these interventions without appropriate evaluation of their useability and effectiveness could lead to the development and promulgation of ineffective, or even harmful, interventions [20]. We therefore present a systematic review and meta-analysis of the characteristics and effectiveness of digital interventions, including both online resources and mobile telephone apps for suicidal ideation and self-harm.

## Methods

The reporting of this systematic review and meta-analysis conforms to the Preferred Reporting Items for Systematic Reviews and Meta-Analyses (PRISMA) guidelines [21].

### Search strategy and selection criteria

We searched for literature indexed in seven electronic databases covering a wide range of disciplines, including: computing and information technology (Applied Science & Technology) clinical trials (Cochrane Central Register of Controlled Trials [CENTRAL]), medicine (Embase; Medline), psychology (PsycARTICLES; PsycINFO), and public health (Global Health) as well as a database that specifically indexes literature on interventions for the prevention of suicide (Centre for Research Excellence in Suicide Prevention [CRESP]). Clinical trial registries were also searched using these same keywords to identify ongoing studies. All databases were searched from their respective start dates until 31 March, 2017.

A two stage process was used to locate relevant studies. At the first stage, keywords inclusive of digital interventions and platforms were combined. Next, using standard Boolean operators, these were combined with keywords related to suicidal ideation and self-harm. There were no restrictions either on publication language or status. For further information on this electronic search strategy, please see the Appendix.

Ancestry searches were also conducted by manually screening the reference lists of included studies and previous reviews [22–26]. Where information on either study design, methodology, or results was either unclear or missing from the published study, we sought clarification from corresponding authors.

#### Selection criteria

Studies were eligible for inclusion if: (1) the effectiveness of a standalone digital intervention (i.e., any online or mobile telephone app) was evaluated; (2) the intervention was designed for the self-management of suicidal ideation or self-harm; (3) data on the effectiveness of the intervention with respect to any suicidal outcome (i.e., suicidal ideation, repetition of self-harm, attempted suicide, or completed suicide) were reported; and (4) either a randomized, pseudo-randomized, or observational pre-test/post-test design was used.

Studies were excluded if: (1) the program was not a standalone digital intervention. Thus multimodal interventions, in which the digital intervention was intended to serve either as a complement or adjunct to traditional face-to-face psychosocial therapy or required significant input or involvement from face-to-face treating clinicians, were not eligible for inclusion in this review. Brief contact-based programs delivered via text messaging or e-mail services were also excluded as no form of active psychosocial therapy is typically provided in the context of these programs. Studies were also excluded if: (2) the intervention targeted gatekeepers (i.e., carers, other health care professionals, or bystanders who may come into contact with suicidal persons); or (3) no data on suicidal ideation, self-harm, attempted and/or completed suicide were reported. Descriptions of programs without data on effectiveness were also excluded.

Two authors (KW and AM) independently screened studies for inclusion. Firstly, titles of all retrieved studies were screened. Next, only studies meeting inclusion criteria following a full text screen were retained. Any disagreements regarding study eligibility were resolved following consensus discussions with the broader group of review authors. Once again, corresponding authors were contacted to request further information on program design, study design, data analysis, or methodology as required.

### Data analysis

Methodological details were extracted from included studies using a standardized extraction form by two authors (KW and AM) working independently of one another. Disagreements were resolved through consensus discussions. Methodological details included: research design, treatment setting, and outcome ascertainment. We also assessed whether those evaluating the intervention were independent from those who developed the intervention.

Data on the primary outcome, suicidal ideation, were also extracted by KW and AM working independently of one another. To make maximal use of the available data, information was extracted irrespective of whether these outcomes were measured continuously, for example as scores on a psychometrically validated measure of suicidal ideation or as numbers of repeat episodes of self-harm, or categorically, as the proportion of participants reporting thoughts of suicide or number of self-harm events. Care was taken, however, to ensure the items used to determine these outcomes were comparable between studies.

Secondary outcomes included: episodes of self-harm, attempted suicide, and completed suicide measured according to self-report and/or hospital or medical records. Where a study reported outcomes at multiple time points, for example at six and 12 months’ follow-up, only data for the longest follow-up period were extracted, in line with recommendations [27].

#### Statistical analysis

Data on the primary outcome (i.e., suicidal ideation) were synthesized using one of two approaches. Where these were reported categorically (e.g., as a difference in proportions reporting thoughts of suicide in the intervention group as compared to the control group), odds ratios (OR) and their accompanying 95% confidence intervals were calculated.

Where outcomes were reported as scores on a continuous scale, such as total scores on the Beck Scale of Suicidal Ideation (BSSI; [28]), the mean difference (MD) or the standard mean difference (SMD), with an accompanying 95% confidence interval, was calculated as appropriate. Specifically, where the same scale was used to investigate the outcome of interest in all studies included in a meta-analysis, the MD was used. The SMD, on the other hand, was used where outcomes were measured using a variety of different scales as recommended [27].

Pooled ORs, MDs, or SMDs were calculated by using the DerSimonian and Laird random effects model [29], as implemented by RevMan for Windows, version 3.5. The impact of between-study heterogeneity was quantified by the *I* ^*2*^ statistic [30]. We interpreted an *I* ^*2*^ statistic of ≥75% as indicating substantial levels of between-study heterogeneity. Where we found evidence of this, we undertook sensitivity analyses to explore potential causes of this between-study heterogeneity, as outlined below.

#### Sensitivity analyses

Given recent findings suggesting that psychological interventions that directly address suicidal ideation and self-harm are more effective in reducing attempted and completed suicide than interventions which indirectly target the symptoms associated with suicidality (e.g., anxiety, depression, hopelessness) [31], we conducted sensitivity analyses to investigate whether digital interventions developed specifically for the self-management of suicidal ideation or self-harm would be more effective in preventing suicidal ideation and repetition of self-harm than interventions developed for the self-management of depression symptomatology more generally.

#### Sub-group analyses

The inclusion of RCTs and non-randomized observational studies within a single meta-analysis is becoming increasingly common as reliance on RCT evidence alone can lead to knowledge translation bias [32]. RCTs, for example, typically recruit highly selected patient populations with lower risk profiles as compared to “real world” populations [33]. The inclusion of results from pre-test/post-test observational studies together with those from RCTs, however, can also lead to over-estimation of the treatment effect size [34]. To balance these two concerns, all studies were eligible for inclusion in our review, irrespective of study design; however, we did not pool data from RCTs together with data from observational studies. Instead, we calculated separate sub-group analyses by study design to investigate what impact, if any, study design had on the magnitude of the effect size observed for these interventions.

### Risk of bias

Risk of bias was assessed using the Cochrane Collaboration tool for randomised and pseudo-randomised controlled trials [35] and, for controlled before/after designed studies, the Risk of Bias In Non-Randomized Studies of Interventions (ROBINS–I; [36]). The Cochrane Collaboration tool assesses bias in seven domains including: adequacy of the random sequence generation, allocation concealment, blinding of participants, clinical personnel, and outcome assessors, as well as incomplete data, selective outcome reporting, and other bias. The ROBINS–I assesses bias in eight domains including: confounding, participant selection, classification of the intervention, departures from the intended intervention, missing data, measurement of outcomes, selection of the reported results, and overall bias.

### Role of the funding source

The funder had no role in study design, data collection, data analysis, data interpretation, or writing of the report. KW and AM had full access to all the data in this study, and all authors had final responsibility for the decision to submit for publication.

## Results

**Fig. 1:**
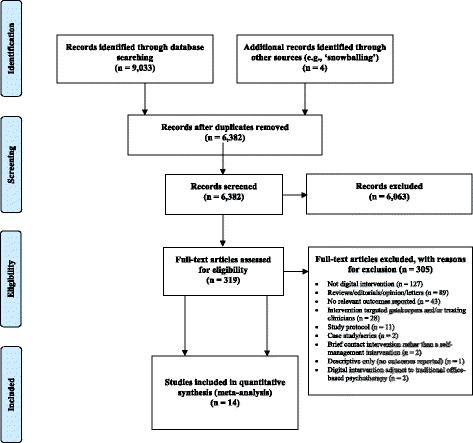
PRISMA flow diagram of included and excluded studies

The electronic search strategy outlined in the Appendix initially identified a total of 9033 potentially relevant records. Four additional records were retrieved following ancestry searching. After excluding duplicates, this figure reduced to 6382 records. Of these, 6063 records were screened out after a review of their titles, whilst 305 records were omitted following full text screening. A total of 14 independent, non-overlapping studies were therefore included in the review, reporting data on a total of 3356 participants (Fig. 1; Table 1).

**Table 1:** Study characteristics, methodological details, and risk of bias assessment for the 14 studies included in this review

Study and Reference Number	Study Design	Country	*N*		Participant Source	Intervention Condition	Control Condition	Measures	Risk of Bias
			*INV*	*CTL*					
Christensen, 2013 [37]	RCT	Australia	38	35	Callers to Lifeline, a charitable 24 h telephone counselling service providing crisis support and suicide prevention. Persons who were highly distressed and/or suicidal at intake were excluded.	*MoodGYM:* Internet self -guided psychoeducation program coupled with self-guided iCBT consis-ting of six modules delivered once a week over a six week period.	Participants were allocated to a wait-list control condition.	*Suicidal ideation:* scores on four suicidal ideation items from the 28-item General Health Questionnaire (GHQ–28)*.*	Neither participants nor clinical personnel were blind to treatment allocation. Proportion completing treatment not stated.
Franklin, 2016 [50]					Recruited from online forums for self-injury. Participants had to self-report two or more episodes of self-cutting in past month to be eligible for participation.	*Therapeutic Evaluative Conditioning:* App-based gamified conditioning program in which self-harm related stimuli were sequentially paired with adverse stimuli. Participants were encouraged to interact with the program as often as necessary over a one month period.	Participants were allocated to an attentional control condition.	*Suicidal ideation:* scores on the Self-Injurious Thoughts and Behaviours Interview, indicating number of days in which the participant had had thoughts of suicide. *Self-harm:* scores on the Self-Injurious Thoughts and Behaviours Interview, indicating the number of days in which the participant had engaged in self-cutting or NSSI.	Neither participants nor clinical personnel were blind to treatment allocation.
Study 1	RCT	International	55	59					
Study 2	RCT	International	62	69					
Study 3	RCT	International	75	84					
Guille, 2015 [41]	RCT	USA	100	99	Medical interns commencing their residency year recruited from one of two hospitals.	*MoodGYM:* Internet self -guided psychoeducation program coupled with self-guided iCBT consis-ting of four modules delivered once a week over a four week period.	Participants were allocated to an attentional control condition.	*Suicidal ideation:* proportion of participants self-reporting any thoughts of suicide according to scores on item nine of the Patient Health Questionnaire–9 (PHQ–9).	Neither participants nor clinical personnel were blind to treatment allocation. One-half (49%) did not complete treatment.
Hedman, 2014 [48]	Pre-test/Post-test	Sweden	1203		Recruited from a university hospital psychia-tric clinic.	*iCBT:* consisting of 10 modules of behavioural activation and cognitive therapy delivered over a 12 week period.	N/A.	*Suicidal ideation:* scores on item 9 of the Montgomery Åsberg Depression Rating Scale (MADRS).	Appropriate adjustment was made for potential time-varying confounding. Neither participants nor clinical personnel were blind to treatment allocation. One-quarter (25%) did not complete treatment.
Hill, 2016 [44]	RCT	USA	40	40	Recruited following an advertising campaign. All participants had to self-report significant levels of perceived burdensomeness (i.e., score of 17 or greater on the Inter-personal Needs Question-naire Perceived Burden-someness sub-scale).	*LEAP:* Internet self-guided program informed by the Interpersonal Theory of Suicide (prin-cipally perceived burden-someness) consisting of two modules delivered over a two week period.	Participants were allocated to psychoeducation.	*Suicidal ideation:* scores on the Beck Scale for Suicidal Ideation.	Participants were not blind to treatment allocation. Control condition did not account for intervention time or engagement (i.e., did not satisfy attentional control requirements). Over one-third (39.0%) did not complete treatment.
Kordy, 2016 [46]	RCT	Germany	77	80	Recruited from one of six psychiatric departments. All patients had to meet diagnostic criteria for major depression accord-ing to the Structured Clinical Intervention for DSM-IV and to self-report a history of at least three prior episodes of depression to be eligible for participation.	*SUMMIT:* Internet self-guided emotion coping program. The number of treatment modules was not stated, but participants were encouraged to access the intervention as often as necessary over a 12 month period.	Participants were allocated to usual psychiatric care, including pharmaco-therapy and/or psychotherapy as necessary.	*Self-harm and/or attempted suicide*: re-hospitalisation following an episode of self-injury and/or a suicide attempt.	Neither participants nor clinical personnel were blind to treatment allocation. Proportion completing treatment not stated.
Mewton, 2015 [38]	Pre-test/Post-test	Australia	484		Patients diagnosed with depression who were prescribed a course of iCBT by their primary care provider.	*iCBT:* Consisting of six modules delivered over six week period.	N/A.	*Suicidal ideation:* scores on item 9 of the Patient Health Questionnaire–9 (PHQ–9).	Appropriate adjustment was made for presence of missing data and for potential time-varying confounding. Neither participants nor clinical personnel were blind to treatment allocation. Almost one-half (43.2%) did not complete treatment.
Mortiz, 2012 [45]	Pseudo-RCT	Germany	105	105	Recruited from websites providing support to persons with depression.	*Deprexis:* self-guided iCBT program consisting of 10 modules delivered over an eight week period.	Participants were allocated to a wait-list control condition.	*Suicidal ideation:* scores on the German language version of the Suicide Behaviors Questionnaire-Revised (SBQ–R).	Neither participants nor clinical personnel were blind to treatment allocation. Participants were alternately allocated to the intervention and control groups. One-fifth (21.9%) did not complete treatment.
Robinson, 2016 [39]	Pre-test/Post-test	Australia	21		Secondary school students presenting to school-based counsellors with suicidal ideation in the past month.	*Reframe-IT:* Internet self-guided iCBT program consisting of eight modules delivered over an eight week period.	N/A.	*Suicidal ideation:* scores on the Suicidal Ideation Questionnaire-Junior (SIQ-JR) or the Adult Suicidal Ideation Questionnaire (ASIQ) (as appropriate to the participant’s age).	Analyses based on treatment completers only (*N =* 21). Neither participants nor clinical personnel were blind to treatment allocation.
Tighe, 2017 [40]	RCT	Australia	31	30	Recruited though a community-based suicide prevention organisation. All participants had to identify as Aboriginal and /or Torres Strait Islander. All participants also had to report clinically sig-nificant levels of depression (i.e., scores of 10 or greater on the Patient Health Questionnaire-9), psychological distress (i.e., scores of 25 or greater on the Kessler Psychological Distress Scale), and had to self-report suicidal thoughts in the past two weeks.	*iBobbly:* App-based self-guided CBT program consisting of three modules completed over a six week period. Partici-pants were also encouraged to complete self-assessments on functioning, suicidal thinking, mind-fulness exercises, self-soothing activities, and cultural engagement activities.	Participants were allocated to a wait-list control condition.	*Suicidal ideation:* scores on the Depressive Symptoms Inventory-Suicidality Subscale (DSI-SS).	Due to changes in inclusion criteria after the commencement of the trial, one-quarter (26.2%) of the included participants did not meet the criterion for frequency of suicidal thoughts. Participants, clinical personnel, and outcome assessors were not blind to treatment allocation. Data on usage was available for 65.6% of the included participants. Of these, 15.0% did not complete treatment.
Van Spijker, 2014 [47]	RCT	Netherlands	116	120	Recruited following an advertising campaign. All participants had to report mild to moderate suicidal ideation to be eligible for participation. Participants self-reporting severe depression were excluded.	*iCBT:* Consisting of six modules delivered over a six week period with added activities from DBT, PST, and mindfulness-based cognitive therapy.	Participants were allocated to an attentional and online bibliotherapy.	*Suicidal ideation:* scores on the Beck Suicide Ideation Scale.	Neither participants nor clinical personnel were blind to treatment allocation. Around one-half (44.0%) did not com-plete at least three treatment modules.
Van Voorhees, 2009 [42]	Pre-test/Post-test^a^	USA	83		Adolescents receiving treatment for depression at primary care facilities registered with one of five major Health Care Organisations (HMOs).	*CATCH-IT:* Internet self-guided program consisting of 14 modules of CBT, IPT, and comm-unity resiliency activities delivered over a 12 week period.	N/A.	*Suicidal ideation:* scores on item 9 of the Patient Health Questionnaire–9 (PHQ–9).	Almost all (96.4%) participants completed treatment. As data were analysed as pre-test/post-test compare-sons in this review, no appropriate adjustment could be made for potential time-varying confounding. Neither participants nor clinical personnel were blind to treatment allocation.
Wagner, 2014 [49]	RCT	Switzerland	32	30	Recruited from university websites, newspapers, poster adverts, and from referrals to a depression self-help group. Participants had to score at least 12 on the Beck Depression Inventory-II at the screening interview.	*iCBT:* Consisting of seven modules delivered over an eight week period.	Participants were allocated to eight weeks of manualised face-to-face CBT delivered by a registered psychologist.	*Suicidal ideation:* scores on the Beck Suicide Ideation Scale.	Neither participants nor clinical personnel were blind to treatment allocation. Despite rand-omisation, a significantly higher number of females were allocated to the intervention. One-fifth (22.0%) did not complete treatment.
Whiteside, 2014 [43]	Pre-test/Post-test	USA	39		Primary care patients receiving treatment for a new episode of depression and who were not already receiving either pharm-acotherapy or psycho-therapy. Those who had received treatment for a depressive episode in the last six months were not eligible to participate.	*Thrive:* iCBT program consisting of three modules delivered over a three week period. Partici-pants were also encouraged to access a secure messaging platform at least once a week for eight weeks to receive a moti-vational intervention from a qualified iCBT coach.	N/A.	*Suicidal ideation:* scores on item 13 of the Symptom Checklist-20.	No appropriate adjustment was made for potential time-varying confounding. Neither participants nor clinical personnel were blind to treatment allocation. One-fifth (22.0%) did not complete treatment.

*Abbreviations*: *CTL* control, *CBT* cognitive behavioural therapy, *iCBT* internet-based cognitive behavioural therapy, *DBT* dialectical behaviour therapy, *DSM-IV* Diagnostic and Statistical Manual of Mental Disorders, fourth revision, *INV* intervention, *IPT* interpersonal therapy, *NSSI* non-suicidal self-injury, *PST* problem-solving therapy, *RCT* randomised controlled trial, *TAU* treatment as usual, *UK* United Kingdom, *USA* United States of America

^a^Although participants were randomised to the intervention and control conditions, all participants in this study received access to the digital intervention. For the purposes of this review, therefore, data from this RCT was treated as pre-test/post-test comparisons instead

### Study characteristics

The majority of these studies had been conducted either in Australia (four studies: [37–40]) or the USA (four studies: [41–44]). Two studies were conducted in Germany [45, 46], one in the Netherlands [47], one in Sweden [48], and one in Switzerland [49]. One further study recruited participants through online forums and therefore included participants from a number of different countries, including countries in North America, Europe, and Australasia [50].

Most studies evaluated the effectiveness of online programs [37–39, 41–49]. Only two evaluated the effectiveness of mobile telephone apps [40, 50]. Most programs were developed for the self-management of depression [37, 38, 42, 43, 45, 46, 48, 49]. However, as these studies assessed the effectiveness of these programs on to at least one suicide-related primary or secondary outcome (i.e., suicidal ideation, self-harm, attempted and/or completed suicide) they were nonetheless eligible for inclusion in this review. Only five programs were developed specifically for the self-management of suicidal ideation [39–41, 44, 47], and only one was developed for the self-management of self-harm [50].

In terms of study design, eight were randomised controlled trials [37, 40, 41, 44, 46, 47, 49, 50], four were observational pre-test/post-test studies [38, 39, 43, 48], and one was a pseudo-randomised controlled trial in which sequential participants were alternately allocated to the intervention and control conditions [45]. For one RCT, although participants were randomised to the intervention and control conditions, all participants received access to the digital intervention [42]. For the purposes of this review, data from this RCT was therefore treated as pre-test/post-test comparisons instead. For two additional RCTs, outcomes at pre-test/post-test were also reported for the intervention group, enabling their inclusion in pre-test/post-test comparisons as well as in RCT comparisons [45, 49]. However, to ensure results from these studies were not double-counted, analyses were pooled separately by study design for all outcomes reported in this review. In one further RCT, participants assigned to the wait-list control condition crossed over to receive the intervention after six weeks [40]. To avoid contamination from any ‘carry-over’ effects, we only extracted data for the first six week period prior to cross-over as recommended [51].

### Types of digital interventions

Most programs were developed by clinical psychologists and/or psychiatrists with experience treating suicidal ideation and/or self-harm [37, 39–45, 47–50], and were evaluated by those who developed the intervention [37–40, 42, 44, 46–50]. In terms of therapeutic approach, most programs were based on the principles of cognitive behavioural therapy (CBT). Some also included elements of ‘third wave’ CBT, such as mindfulness [45], dialectical behaviour therapy [47], or mentalisation-based cognitive therapy [47]; all of which hold the rationale that challenging thoughts, a principle feature of CBT, is less important than understanding and accepting thoughts in a non-judgemental manner. Other programs included a variety of treatment approaches, including: acceptance-based therapy [40], problem-solving therapy [47], interpersonal therapy [42], mood monitoring [46], and crisis planning [46]. Only one program utilised gamification in which participants were presented with a series of visual stimuli pairs designed to condition aversive reactions to self-harming thoughts or behaviours [50].

### Types of control conditions

For the eight RCTs and one pseudo-RCT designed trials, the interventions were compared against a number of types of control conditions, including: wait-list control [37, 40, 45], attentional control [41, 47, 50], psychoeducation [44], treatment as usual [46], or face-to-face psychotherapy [49].

### Ongoing studies

An additional 10 ongoing studies were identified at the time of the systematic search [52–59]. Further details of these ongoing trials are reported in Table 2.

**Table 2:** Study characteristics, methodological information, and trial registration information for the 10 ongoing studies identified by this review

Study and Reference Number	Study Design	Country	Target *N*		Participant Source	Intervention Condition	Control Condition	Measures	Trial Registration Number
			*INV*	*CTL*					
Boele, 2014 [52]	RCT	Netherlands	63	63	Adults with grade II, III, or IV glioma (assessed according to WHO criteria) scoring 12 or greater on the Center for Epidemiological Studies De-pression Scale.	Internet self-guided problem solving therapy consisting of five modules delivered once a week over a five week period.	Waitlist.	*Suicidal ideation:* scores on the Beck Scale for Suicidal Ideation (BSSI).	Netherlands Trial Register: NTR3223.
Eylem, 2015 [53]	RCT	Netherlands and UK	100	100	Community-dwelling adults either born in Turkey, or with one or both parents born in Turkey with a score of one or greater on the Beck Suicidal Ideation Scale.	*iCBT:* Consisting of between six and eight modules delivered once a week over a six week period.	Waitlist.	*Suicidal ideation:* scores on the Beck Scale for Suicidal Ideation (BSSI) and the Suicidal Ideation Attribute Scale (SIAS).	Not stated.
Gumpert, 2016 [Not Published]	Pre-test/Post-test	Sweden	43		Adolescents diagnosed with NSSI, defined according to section three of the DSM-5, with at least one episode of NSSI occurring within the past month.	Internet emotion regulation training. No information on the number of modules or the duration of therapy were provided.	N/A.	*Self-Harm:* self-reported self-harm according to the Deliberate Self-Harm Inventory (DSHI). *Suicidal ideation:* scores on the Suicidal Ideation Questionnaire-Junior (SIQ-JR).	Clinical Trials Register: NCT02697019.
Kaslow, 2016 [Not Published]	Pre-test/Post-test	USA	25		Referrals for behavioural health treatment through an outpatient mental health service following a suicide attempt or clinically significant suicidal ideation.	*RefliefLink:* App-based mood tracking and stress management intervention consisting of modules of relaxation exercises, guided meditation, progressive re-laxation, and mindfulness skills training. No information on the number of modules or the duration of therapy were provided.	N/A.	*Suicidal ideation and behaviours:* measured according to scores on the Columbia Suicide Severity Rating Scale Screener (C-SSRS) as well as the number of participants self-reporting episodes of self-injury.	Clinical Trials Register: NCT02691221.
Mühlmann, 2017 [54]	RCT	Denmark	175	175	Community-dwelling adults scoring three or greater on the Beck Scale for Suicidal Ideation.	*iCBT:* Consisting of six modules delivered once a week over a six week period.	Waitlist.	*Suicidal ideation:* scores on the Beck Scale for Suicidal Ideation (BSSI) and the Suicidal Ideation Attribute Scale (SIAS). *Self-Harm:* number of participants with a hospital and/or medical record for self-harm. *Suicide:* number of suicide deaths according to a national mortality register.	Clinical Trials Register: NCT02872610.
Perry, 2015 [55]	cRCT	Australia	1600 (30 schools)		Community-dwelling adoles-cents enrolled in their final year of high school.	*SPARX-R:* Internet gamified iCBT program consisting of seven modules delivered once a week over seven weeks.	Attention placebo.	*Suicidal ideation and/or attempted suicide:* scores on the suicidal thoughts, plans, and attempted suicide items of the Youth Risk Behaviour Survey (YRBS).	Australian and New Zealand Clinical Trials Register: ACTRN12614000316606.
Robinson, 2014 [56]	cRCT	Australia	170 (28 schools)		Community-dwelling adoles-cents enrolled in high school self-reporting any suicidal ideation over the past month.	*Reframe-IT:* iCBT program consisting of eight modules delivered over a 10 to 12 week period.	TAU.	*Suicidal ideation:* scores on the Suicidal Ideation Questionnaire (SIQ). *Self-Harm and Attempted Suicide:* scores on a specifically-developed instrument.	Australian and New Zealand Clinical Trials Register: ACTRN12613000864729.
Simon, 2016 [57]	Zelen RCT	USA	>2500		Referrals to an outpatient mental health and/or general medical centre who report daily or almost daily suicidal ideation according to scores on item 9 of the Patient Health Questionnaire-9.	Internet DBT with a specific focus on the development of mindfulness skills, non-judgemental acceptance of emotions, opposite action, and paced breathing. This program will consist of four self-paced modules.	TAU.	*Suicidal ideation:* scores on a specifically developed six-item version of the Columbia Suicide Severity Rating Scale Screener (C-SSRS).	Clinical Trials Register: NCT02326883.
Stallard, 2016 [58]	Pre-test/Post-test	UK	50		Community-dwelling adoles-cents with a history of repeated self-harm who are currently receiving ongoing care in out-patient child and adolescent mental health services.	*BlueIce:* App-based toolbox, developed in consultation with young people who self-harm, including a mood diary, mood-lifting exercises drawn from CBT and DBT, physical activities, and mindfulness exercises. Part-icipants are also encouraged to upload and share inspirational photos and music files. Participants will use BlueIce daily for 10 weeks.	N/A.	*Self-Harm*: measured according to self-report, and GP report, and/or medical records.	Not stated.
Van Spijker, 2015 [59]	RCT	Australia	570		Community-dwelling adults reporting current suicidal ideation according to item five of the Columbia-Suicide Severity Rating Scale, but who do not self-report attempting suicide within the past month.	*iCBT:* Consisting of six modules delivered once a week over a six week period.	Attention placebo.	*Suicidal ideation:* scores on the Intensity of Suicidal Ideation sub-scale of the Columbia Suicide Severity Rating Scale Screener (C-SSRS) and the Suicidal Ideation Attribute Scale (SIAS).	Australian and New Zealand Clinical Trials Register: ACTRN12613000410752.

*Abbreviations*: *CTL* control, *CBT* cognitive behavioural therapy, *iCBT* internet-based cognitive behavioural therapy, *cRCT* cluster randomised controlled trial, *DBT* dialectical behaviour therapy, *DSM-IV* Diagnostic and Statistical Manual of Mental Disorders, fourth revision, *DSM-5* Diagnostic and Statistical Manual of Mental Disorders, fifth revision, *GP* general practitioner, *INV* intervention, *IPT* interpersonal therapy, *NSSI* non-suicidal self-injury, *PST* problem-solving therapy, *RCT* randomised controlled trial, *TAU* treatment as usual, *UK* United Kingdom, *USA* United States of America

### Suicidal ideation

**Fig. 2:**
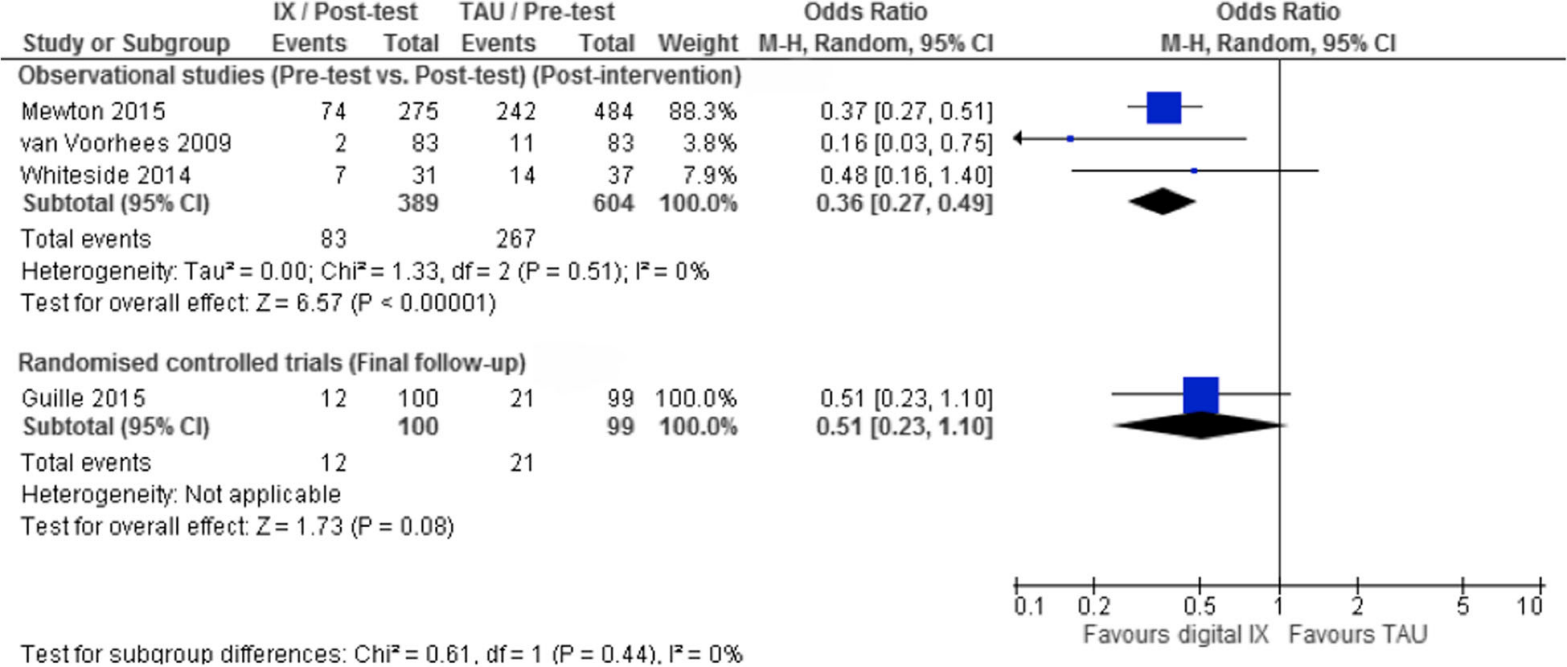
Random effects odds ratio (OR) and accompanying 95% confidence interval (CI) for digital interventions on the proportion of participants reaching defined clinical thresholds for suicidal ideation

Four studies reported data on the number of participants self-reporting suicidal ideation. At post-intervention, there was some suggestion of a reduction in the proportion of participants self-reporting suicidal ideation in three observational pre-test/post-test studies (OR 0.36, 95% CI 0.27 to 0.49, 3 studies, *I* ^*2*^ = 0%, *p* < 0.0001; Fig. 2). However, by the final follow-up assessment, data from one RCT suggested no evidence of a treatment effect for these interventions (Fig. 2). There was no evidence of a significant difference in the magnitude of the treatment effect by study design for this outcome (χ^2^ = 0.61, *df* = 1, *p* = 0.44). As all four studies included in this analysis investigated the effectiveness of digital interventions specifically developed for the self-management of depression symptoms (rather than suicidal ideation or self-harm specifically), sensitivity analyses could not be undertaken.

**Fig. 3:**
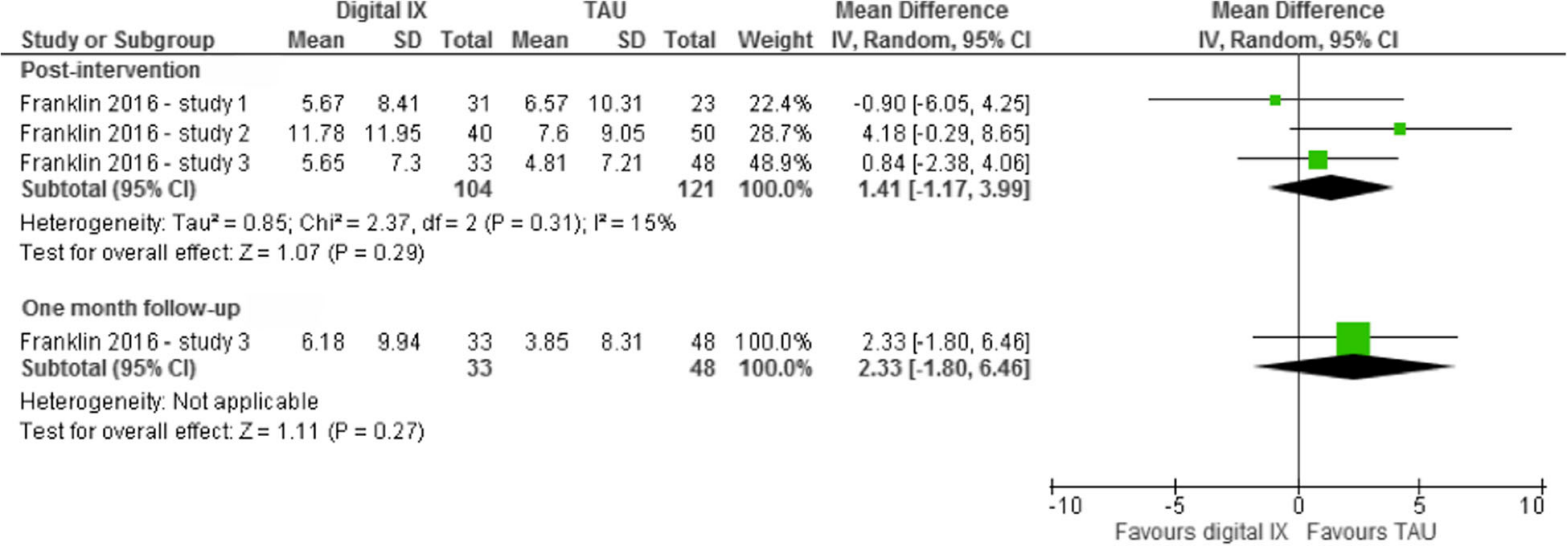
Random effects mean difference (MD) and accompanying 95% confidence interval (CI) for digital interventions on frequency of self-reported suicidal ideation

One study covering three related RCTs reported information on the frequency of self-reported episodes of suicidal ideation at post-intervention (for all three studies) and at the conclusion of the final follow-up assessment (for one of these three studies) [50]. However, no evidence of a treatment effect was found for these interventions at either time point (Fig. 3). As only one RCT was included in this analysis, neither tests for subgroup differences nor sensitivity analyses could be undertaken.

Eight studies reported data on suicidal ideation scores using a number of different psychometric instruments, including: the BSSI [44, 47, 49], the Depressive Symptom Inventory–Suicidality Subscale (DSI-SS; [60]) [40], the Suicide Behaviors Questionnaire–Revised (SBQ–R; [61]) [45], the Suicidal Ideation Questionnaire–Junior (SIQ–J; [62]) [39], the four item suicidal ideation sub-scale of the General Health Questionnaire (GHQ–28; [63]) [37], and the suicidal ideation item of the Hopkins Symptom Checklist (HSCL–20; [64]) [43]. As raw means and standard deviations were not reported for suicidal ideation scores in one RCT, data were instead estimated from graphics in the original report for this study [37].

**Fig. 4:**
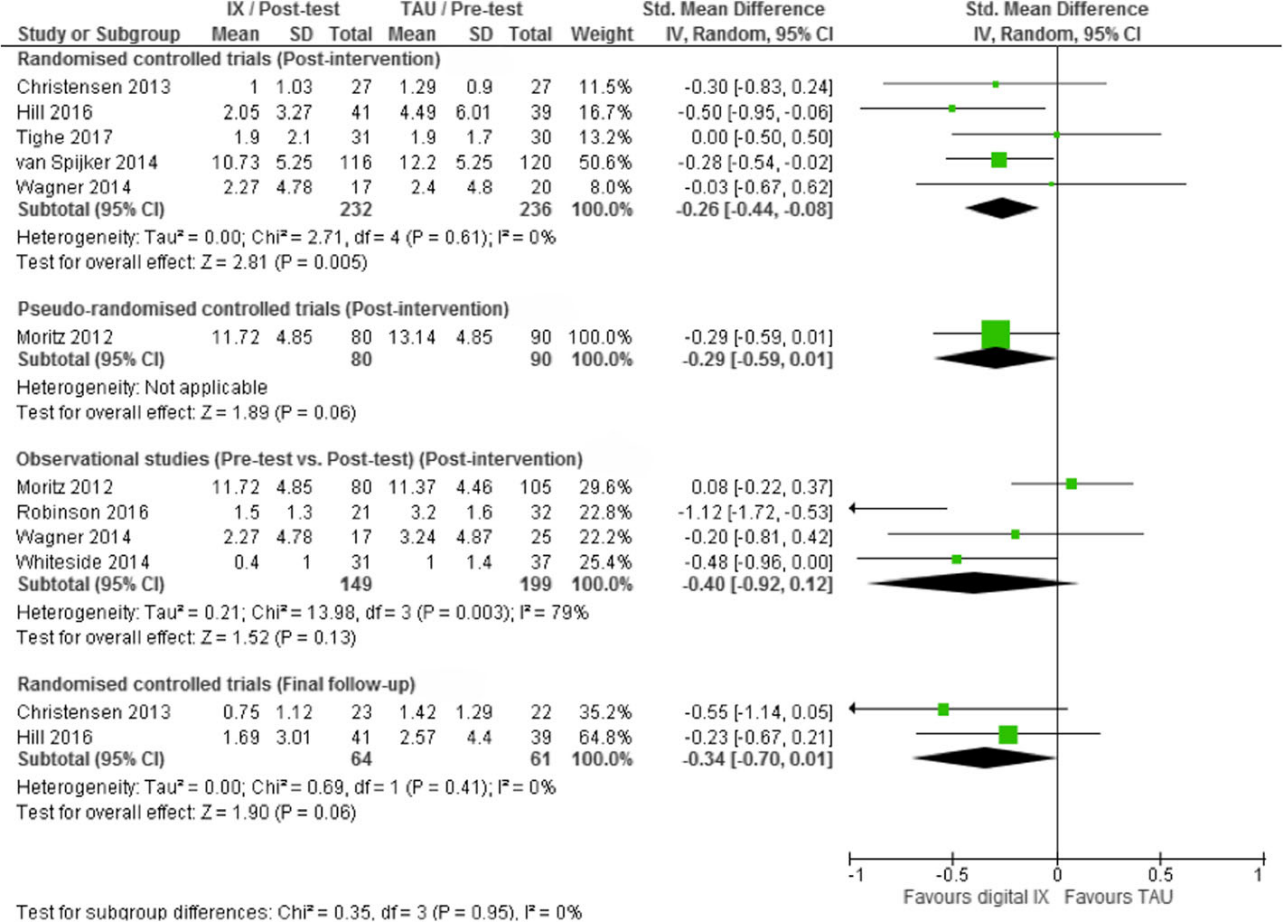
Random effects standard mean difference (SMD) and accompanying 95% confidence interval (CI) for digital interventions of suicidal ideation scores

At post-intervention, there was evidence these interventions were associated with a significant reduction in suicidal ideation scores in five RCTs (SMD -0.26, 95% CI -0.44 to −0.08, 5 studies, *I* ^*2*^ = 0.0%, *p* = 0.005; Fig. 4). There was no evidence of a significant treatment effect for these interventions in either one pseudo-randomised controlled trial or four observational studies, however (Fig. 4). The test for subgroup differences was non-significant suggesting there was no difference in magnitude of the effect size by study design for this outcome (χ^2^ = 0.25, *df* = 2, *p* = 0.88). Sensitivity analyses including only those studies in which the intervention was specifically developed for the self-management of suicidal ideation or self-harm also did not materially affect these results (results not shown).

Two RCTs reported data on suicidal ideation at the final follow-up assessment [37, 44]. For one of these trials, however, data on suicidal ideation scores at final follow-up had to be estimated by review authors from graphics presented in the original trial report [37]. Data from these trials suggested these digital interventions were not associated with a significant treatment effect for suicidal ideation by this time point (SMD -0.34, 95% CI –0.70 to 0.01, 2 studies, *I* ^*2*^ = 0.0%, *p* = 0.06; Fig. 4). Given that both studies included in this analysis were RCTs, neither tests for subgroup differences nor sensitivity analyses could be undertaken. One further RCT presented data on outcomes at final follow-up for the intervention group only [65]. Analyzing these data as pre-test/post-test comparisons suggested a significant reduction in suicidal ideation scores at the final follow-up assessment in this trial (MD -4.90, 95% CI -7.07 to −2.73, 1 study, *I* ^2^ = not applicable, *p* < 0.001). As only one RCT reported data at the final follow-up assessment, neither tests for subgroup differences nor sensitivity analyses could be undertaken.

### Self-harm

**Fig. 5:**
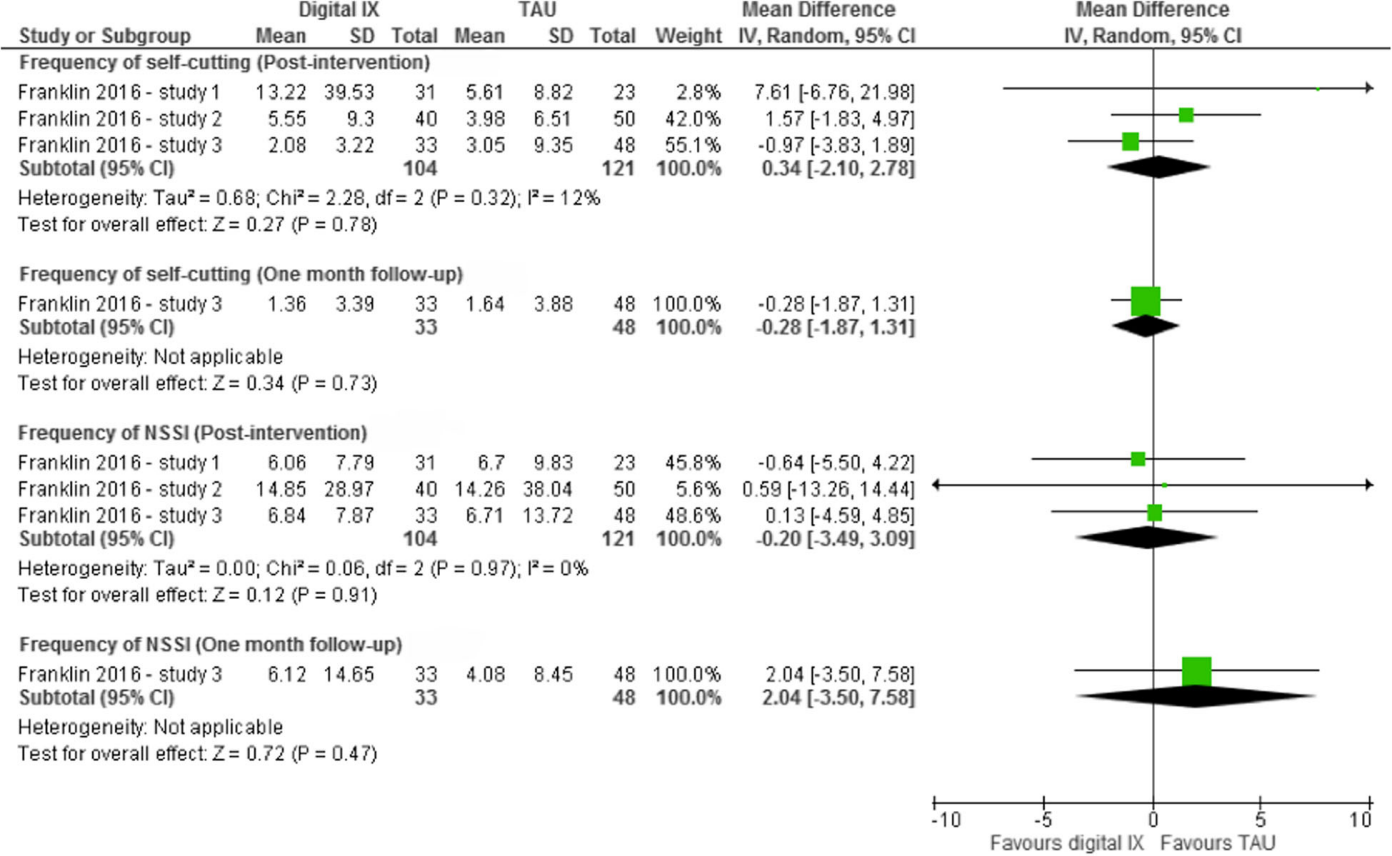
Random effects mean difference (MD) and accompanying 95% confidence interval (CI) for digital interventions on frequency of self-reported self-cutting and non-suicidal self-injury (NSSI)

One study, covering three related RCTs, evaluated the effectiveness of digital interventions on frequency of self-harm episodes [50]. At post-intervention, there was no indication of a treatment effect for these interventions on either self-reported frequency of self-cutting or non-suicidal self-injury in these three RCTs (Fig. 5). There was also no indication of a treatment effect for this intervention on frequency of self-reported self-cutting or non-suicidal self-injury at the final follow-up assessment (at one month) in one of these RCTs (Fig. 5). Sensitivity analyses could not be undertaken for this outcome as only one study investigated outcomes relating to repetition of self-harm. As all three studies were RCTs, subgroup analyses to investigate the impact of study design on the magnitude of the effect size could also not be undertaken.

### Combined self-harm and attempted suicide

One RCT evaluated the effectiveness of a digital intervention on attempted suicide and self-harm [46]. As results for self-harm could not be disaggregated from that for attempted suicide, they are instead analysed here as a combined outcome. There was no evidence of a reduction in the proportion of participants who attempted suicide and/or engaged in self-harm over a 24 month follow-up period in this study (OR 2.11, 95% CI 0.19 to 23.81, 1 study, *I* ^*2*^ = not applicable, *p* = 0.55). Once again, as only one study investigated outcomes relating to combined attempted suicide and/or self-harm, sub-group and sensitivity analyses could not be undertaken.

### Attempted suicide

One RCT evaluated the effectiveness of a digital intervention on attempted suicide [47]; however, no evidence of a reduction in the proportion of participants self-reporting a suicide attempt was noted by the post-intervention assessment in this study (OR 0.58, 95% CI 0.16 to 2.02, 1 study, *I* ^*2*^ = not applicable, *p =* 0.39). As only one study investigated outcomes relating to combined attempted suicide, sub-group and sensitivity analyses could not be undertaken.

## Discussion

This systematic review and meta-analysis evaluated the effectiveness of digital interventions, including both online and mobile telephone applications (‘apps’) for the self-management and/or treatment of suicidal ideation or behaviours. A total of 14 studies were included in the present review, reporting data for a total of 3356 participants.

Overall, this review found some evidence that digital interventions may be associated with reductions in suicidal ideation, particularly at the post-intervention assessment. It is notable, however, that where these interventions were associated with significant treatment benefits for suicidal ideation, these effects tended to be stronger in observational pre-test/post-test-designed studies as compared with RCTs. Most of the included studies conducted to date, however, have utilised a pre-test/post-test observational design. There was no evidence to suggest these interventions are associated with reductions in self-harm or attempted suicide, although only three studies investigated these outcomes [46, 47, 50]. Few of these studies would have been adequately powered to evaluate rare outcomes, including repetition of self-harm and suicide reattempts.

Adherence was poor in majority of these studies; although this was not clearly related to the number of treatment modules. Of those studies that reported information on adherence [38–50], for example, up to one-half of participants allocated to the intervention group did not complete all treatment modules. Adherence during the long-term follow-up period, which was reported in one RCT [50], was also poor; over one-half (64%) of participants allocated to the intervention group in this trial did not access the intervention at all during the one month follow-up period in this RCT [50]. This suggests that the use of digital innovations, including gamification, may be insufficient to keep these interventions engaging over the longer term.

### Limitations of the included studies

Most studies were of low to moderate quality, as assessed using the Cochrane Collaboration’s tool for randomised and pseudorandomised controlled trials [35], or the ROBINS–I tool for controlled pre-test/post-test studies [36], with biases most apparent for the domains of participant, clinical personnel, and outcome assessor blinding. Performance and detection bias therefore cannot be ruled out.

Of those studies utilising an RCT design, most compared the intervention to either waitlist control [37, 45] or attentional control [41, 47, 50] conditions. Variability in the control condition has been found to be associated with the magnitude of the treatment effect in office-based psychosocial interventions for depression and anxiety [66, 67]. Yet, despite this, variability in the control condition is a rarely investigated source of heterogeneity in meta-analyses of digital interventions.

The included studies report data on a variety of different outcomes. Although all studies included at least one suicidal ideation and/or behaviour relevant outcome, these were measured in a variety of different ways, including from psychometric scales, self-report, or according to hospital or medical records. Given that these outcomes were measured using the same methodology within individual studies, the effect of outcome measure definition on case identification at the post-intervention and follow-up assessments could be expected to affect the intervention and control groups equally. Between studies, however, outcome measure definition may have affected our results in light of research findings suggesting that self-reported self-harm underestimates hospital-treated self-harm [68]. We were unable to assess whether variability in the magnitude of the treatment effect size was related to the way in which the outcome measure was assessed in this review, however, owing to the small number of studies included in any one meta-analysis. Further work will be necessary to pick apart the influence of outcome definition on the apparent effectiveness of interventions, including digital interventions, for the prevention of self-harm.

Most studies (71.4%) also reported information on depression [38–40, 42–45, 47–49]. Fewer studies reported data on other clinically relevant outcomes, such as hopelessness [40], and none reported information on problem-solving following the completion of the intervention. The National Institute for Health and Clinical Excellence (NICE) guidelines recommend that all interventions for self-harm should, at a minimum, investigate the effect of psychological interventions for self-harm on potential mechanisms of action, including depression, hopelessness, and problem-solving [69], echoing more recent calls to this effect in the international scholarship [70, 71]. Online resources may normalize self-harm and may provide vulnerable individuals with access to self-harm content and imagery, including information on methods of self-harm [72]. Additionally, digital interventions that provide some form of active psychosocial therapy, and particularly those that include a focus on mood monitoring, may lead to increased negative affectivity and rumination [73]. Outcomes relating to negative affect and rumination should therefore also be reported for all evaluations of digital interventions for this patient group in future.

Most programs (57.1%) were developed for the self-management of depression [37, 38, 42, 43, 45, 46, 48, 49]. Recent findings for psychosocial interventions, however, suggest that those developed specifically for the management of suicidal thinking are associated with greater impacts for the reduction of attempted and completed suicide as compared to those targeting indirect symptoms associated with suicidal behaviour, such as anxiety, depression, or hopelessness [31]. Future studies in this area should evaluate the degree to which these digital interventions lead to meaningful change in the proposed mechanism(s) of action in order to identify the treatment module(s) associated with the greatest impacts in reducing suicidal ideation and self-harm in this population.

The included studies also examined interventions of varying intensity. Whilst it could be expected that treatments of greater intensity will have greater impacts on reducing suicidal ideation and self-harm, a recent meta-regression review found no evidence to suggest that treatment intensity, measured as the total number of available treatment sessions, was associated with greater effectiveness for office-based psychosocial therapy for self-harm repetition [74].

Finally, it is also likely that these populations will have a very low risk for suicidal behaviour as all studies recruited participants from the community. Despite this, almost all (78.6%) studies included indicated samples, such as callers to telephone or online counselling services [37, 45, 50], or those already in contact with primary care [38, 42, 43], counselling [39, 40, 49] or psychiatric services [46, 48].

### Strengths and limitations of the present review

The majority of these interventions were based on the principles of standard cognitive behavioural therapy (CBT), which has been found to have efficacy in reducing repetition of self-harm in clinical populations in a recent systematic review of psychosocial interventions for the treatment of self-harm [10]. The findings of the present review significantly extend this by suggesting that digital interventions which incorporate the principles of standard CBT may have promising effects in reducing suicidal ideation in non-clinical populations, at least in the short-term.

Whilst we utilized a comprehensive search to locate all relevant trials of digital interventions for the self-management of self-harm, we identified only two mobile telephone apps [40, 50]. Given that a recent Australian study identified a total of 24 apps for the prevention of suicidal behaviour are currently available for download from the Australian Google Play and iOS store [75], this would suggest that a large number of these apps have no evidence to support their effectiveness. It is likely that a similar proportion of online interventions would have little evidence to support their effectiveness.

## Conclusions

Although a growing number of both online and mobile telephone applications (‘apps’) for the self-management and treatment of suicidal thinking and behaviours are now available, few of these have been evaluated for their effectiveness in reducing these outcomes. We identified just 14 in this review. Overall, while there is some promise of these interventions to reduce suicidal ideation, how this translates into reductions in self-harm and/or attempted suicide is unclear at present. Given the prevalence of suicidal ideation in clinical populations, additionally, it is unclear whether these reductions would be clinically meaningful at present.

## Appendix

### Electronic Search Strategy for the Medline and the Cochrane Library

Provides keywords used and electronic search strategy for the Medline and the Cochrane Library. Current to 31 March, 2017.

**Electronic search strategy**

**Table 3:** Cochrane Library

Keyword		Number of ‘hits’
#1	‘mhealth’	4
#2	‘ehealth’	2
#3	web	790
#4	online	178
#5	internet	126
#6	‘mobile device*’	10
#7	‘mobile phone’	23
#8	‘cell* phone’	18
#9	smartphone	4
#10	‘phone app*’	39
#11	‘mobile app*’	36
#12	‘cell* app*’	1134
#13	‘tablet computer*’	6
#14	ipad*	0
#15	iphone*	0
#16	samsung	0
#17	android	0
#18	‘windows phone*’	0
#19	#1 OR #2 OR #3 OR #4 OR #5 OR #6 OR #7 OR #8 OR #9 OR #10 OR #11 OR #12 OR #13 OR #14 OR #15 OR #16 OR #17 OR #18	2007
#20	‘auto$mutilat*’	0
#21	‘self$harm’	19
#22	‘self$injur*’	11
#23	‘self$mutilat*’	1
#24	‘self$cutt*’	0
#25	‘self$poison*’	5
#26	‘drug overdos*’	16
#27	overdos*	22
#28	‘self$destructive behavio*’	0
#29	‘suicid* attempt*’	14
#30	suicid*	69
#31	#20 OR #21 OR #22 OR #23 OR #24 OR #25 OR #26 OR #27 OR #28 OR #29 OR #30	102
#32	#19 AND #31	32

*Dates searched:* Start Date to 30.06.2016

**Table 4:** MEDLINE (OVID interface)

Keyword		Number of ‘hits’
#1	‘mhealth’.mp	741
#2	‘ehealth’.mp	1199
#3	web.mp	61,883
#4	online.mp	56,082
#5	internet.mp	76,766
#6	‘mobile device*’.mp	1159
#7	‘mobile phone’.mp	2999
#8	‘cell* phone’.mp	1540
#9	smartphone.mp	2325
#10	‘phone app*’.mp	229
#11	‘mobile app*’.mp	2291
#12	‘cell* app*’.mp	19,603
#13	‘tablet computer*’.mp	340
#14	ipad*.mp	696
#15	iphone*.mp	400
#16	samsung,mp	527
#17	android.mp	1057
#18	‘windows phone*’.mp	11
#19	#1 OR #2 OR #3 OR #4 OR #5 OR #6 OR #7 OR #8 OR #9 OR #10 OR #11 OR #12 OR #13 OR #14 OR #15 OR #16 OR #17 OR #18	186,347
#20	‘auto?mutilat*’.mp	123
#21	suicide, attempted/ or self-injurious behavior/or ‘self?harm’.mp.	22,723
#22	suicidal ideation/ or suicide, attempted/ or suicide/or suicid*.mp.	75,063
#23	overdos*’.mp	16,949
#24	#20 OR #21 OR #22 OR #23	92,822
#25	#19 AND #24	1265

*Dates searched:* Start Date to 31.03.2017

## Abbreviations

App: Application
BDI–II: Beck Depression Inventory, version two
BSSI: Beck Scale of Suicidal Ideation
CBT: Cognitive behavioural therapy
CDRS–R: Children’s Depression Rating Scale–Revised
CENTRAL: Cochrane Central Register of Controlled Trials
CES–D: Center for Epidemiologic Studies Depression scale
CI: Confidence Interval
CRCT: Cluster randomised controlled trial
CRESP: Centre for Research Excellence in Suicide Prevention
DBT: Dialectical behaviour therapy
DSI–SS: Depressive Symptom Inventory-Suicidality Subscale
DSM-5: Diagnostic and Statistical Manual of Mental Disorders, fifth revision
GHQ–28: General Health Questionnaire, 28 item
HSCL–20: Hopkins Symptom Checklist, 20 item
MADRS: Montgomery-Åsberg Depression Rating Scale
ME: Mean difference
OR: Odds ratio
PHQ–9: Patient Health Questionnaire, 9 item
PRISMA: Preferred Reporting Items for Systematic Reviews and Meta-Analyses
RADS–2: Reynolds Adolescent Depression Scale-Version 2
RCT: Randomised controlled trial
ROBINS–I: Risk of Bias in Non-Randomized Studies of Interventions
SBQ–R: Suicide Behaviors Questionnaire-Revised
SIQ–J: Suicidal Ideation Questionnaire-Junior
SMD: Standard mean difference
TAU: Treatment as usual
UK: United Kingdom
USA: United States of America
WHO: World Health Organization
